# Response to Letter to the Editor from Anthanont Pimjai: Emerging Markers of Atherosclerosis Before and After Bariatric Surgery

**DOI:** 10.1007/s11695-015-1577-y

**Published:** 2015-01-21

**Authors:** Justyna Domienik-Karłowicz, Wojciech Lisik, Zuzanna Rymarczyk, Olga Dzikowska-Diduch, Andrzej Chmura, Urszula Demkow, Piotr Pruszczyk

**Affiliations:** 1 Department of General Medicine and Cardiology, Medical University of Warsaw, Lindley’a 5, 02-005 Warsaw, Poland; 2 Department of General Surgery and Transplantology, Medical University of Warsaw, Nowogrodzka 59, 02-005 Warsaw, Poland; 3 Department of Laboratory Medicine and Clinical Immunology of Developmental Age, Medical University of Warsaw, Marszałkowska 24, 00-576 Warsaw, Poland

Dear Dr. Anthanont Pimjai,

We have carefully read with great interest your letter to the Editor with comments and concerns about the results of our study [1]. We would like to make some remarks.

All women included in our study, both in the study and in the control group, were Caucasian [2]. Therefore, the study group was homogeneous in terms of ethnicity, so no ethnic variations could be observed.

However, we agree with you that the available data on the potential influence of adiponectin on cardiovascular disease is rather equivocal. Aung et al. in their meta-analysis (14,063 CVD patients enrolled) showed a strong positive association of adiponectin with cardiovascular mortality (*n* = 11 studies, overall pooled effect estimate = 1.69 [1.35–2.10]) [3]. That was in accordance with a study by Schondorf et al. as well as our research [2, 4].

Furthermore, it is worth noticing that some studies have suggested that high-molecular-weight adiponectin is a stronger risk factor for cardiovascular diseases than total adiponectin level [5]. In another study, serum adiponectin levels were found to be inversely correlated with intima-media thickness as a marker of carotid atherosclerosis [6].

As Dr Pimjai mentioned before, other adequately powered studies failed to identify adiponectin as a cardiovascular risk factor.

Some researchers claim that these contradictory results in this field might be caused by confounding factors, sex and age of the analyzed subjects, different oligomers of adiponectin tested, other methodological issues (various assays: radioimmunoassays or ELISAs), or handling of laboratory samples.[7]

We agree with Dr Pimjai that further, well-designed studies are needed to clarify the full relationship between adiponectin and the risk of cardiovascular disease.
